# HMGB1 orchestrates STING-mediated senescence via TRIM30α modulation in cancer cells

**DOI:** 10.1038/s41420-021-00409-z

**Published:** 2021-02-08

**Authors:** Je-Jung Lee, In Ho Park, Man Sup Kwak, Woo Joong Rhee, Songhee H. Kim, Jeon-Soo Shin

**Affiliations:** 1 Department of Microbiology, Yonsei University College of Medicine, Seoul, South Korea; 2 Institute for Immunology and Immunological Diseases, Yonsei University College of Medicine, Seoul, South Korea; 3 Severance Biomedical Science Institute, Yonsei University College of Medicine, Seoul, South Korea; 4 Brain Korea 21 Project for Medical Science, Yonsei University College of Medicine, Seoul, South Korea; 5 Center for Nanomedicine, Institute for Basic Science (IBS), Yonsei University, Seoul, South Korea

**Keywords:** Cancer immunotherapy, Cancer prevention

## Abstract

Although cellular senescence has emerged as a novel therapeutic concept in cancer, its underlying mechanisms remain unclear. High mobility group box 1 (HMGB1) and stimulator of interferon genes (STING) are involved in senescence. However, their interactions in senescence have not been reported. Therefore, in this study, we investigated the relationships between HMGB1 and STING in senescence in cancer and other cells. In mouse melanoma cells and several other cell lines, doxorubicin treatment induced senescence in an HMGB1-dependent manner. These responses were mediated by STING, and this function of STING was negatively regulated by the E3 ligase tripartite motif protein 30α (TRIM30α). We also found that HMGB1 bound to the TRIM30α promoter and then suppressed its expression by inhibiting its transcription, which enhanced STING-induced senescence. This mechanism was further mediated by signal transducer and activator of transcription 6 (STAT6) and p21. Overall, our findings demonstrated that HMGB1 orchestrated STING-STAT6-p21-mediated senescence by regulating TRIM30α as an alternative anticancer mechanism.

## Introduction

Senescence is characterized by permanent cell cycle arrest and can be induced in multiple primary cell types following DNA damage, such as global reorganization and degeneration of chromatin, as is observed in various human diseases, including cancer and aging^1^. Senescent cells show the activity of senescence-associated β-galactosidase (SA-β-GAL); expression of tumor suppressors and cell cycle inhibitors, such as retinoblastoma protein, p53, p16, p27, and p21; and lack of proliferative markers. Moreover, these cells typically also show expression of DNA damage markers; nuclear foci of constitutive heterochromatin, which are marked with γ-H2AX and H3K9 trimethylation; and abundant secretion of signaling molecules, such as interleukin (IL)-6 and IL-8^2,3^.

Loss of nuclear lamin B1 protein, another senescence marker, disrupts the integrity of the nuclear envelope. Chromatin fragments from the nuclear membrane bleb out to the cytoplasm and become cytoplasmic chromatin fragments (CCFs)^4^. The cytosolic DNA sensor cyclic GMP-AMP synthase (cGAS) then recognizes CCFs and generates cytosolic GMP-AMP, which activates the stimulator of interferon genes (STING)^5^. The cGAS/STING pathway is crucial to various cancer therapies, including chemotherapy, radiation therapy, and immunotherapy, and is also linked to inflammation and senescence. In a mouse model, significantly fewer gray hairs were observed in STING-null mice after IR than in control mice^4–6^. Furthermore, the responses of cancer cells to stresses are varied, and cancer therapy has been shown to induce senescence and activate the cGAS/STING pathway^7^.

As a member of the highly conserved nonhistone DNA-binding high-mobility group (HMG) protein family, HMG box 1 (HMGB1) protein shows rapid electrophoretic mobility^8^ and is located in the nuclei in most cells. HMGB1 has roles as a DNA chaperone, functioning to maintain nuclear homeostasis, and a danger-associated molecular pattern molecule, acting alone or with endogenous or exogenous pathogen-associated molecular pattern molecules to induce inflammation^9–12^.

Although HMGB1 has been shown to have important roles in cancer, the results of various studies have been contradictory. For example, in some studies, HMGB1 has been shown to promote cancer development and chemotherapy resistance; however, in other studies, HMGB1 exhibits tumor-suppressive activities^13^. HMGB1 is also associated with senescence^14–17^, although its role in cGAS/STING-dependent senescence in cancer cells has not been determined.

Therefore, in this study, we evaluated the role of HMGB1 and its relationship with the STING pathway in genotoxic stress-induced senescence in cancer cells. Our results provide insights into the function of HMGB1 in STING-dependent senescence, which could be applicable to cancer prevention.

## Materials and methods

### Cell culture, transfection, and reagents

B16 and B16-F10 mouse melanoma cells, mouse embryonic fibroblasts (MEFs), J774 mouse macrophages, and HEK293T cells were cultured in Dulbecco’s modified Eagle’s medium. The medium was supplemented with 10% fetal bovine serum (Life Technologies, Waltham, MA, USA) and 1% penicillin–streptomycin (Life Technologies). The Myc-tagged *HMGB1* gene was inserted into the pCMV plasmid for mammalian cell expression. The E3 ligase tripartite motif protein 30α (TRIM30α) promoter was inserted into the pGL3 Luciferase Reporter vector (Promega, Madison, WI, USA). STING and TRIM30α plasmids were purchased from Addgene (Watertown, MA, USA). Plasmid and siRNA transfections were carried out using FuGene HD reagent (Promega) and RNAiMAX (Invitrogen, Carlsbad, CA, USA) respectively, as recommended by the manufacturers.

Anti-HMGB1 (cat. no. ab79823), anti-TRIM30α (cat. no. ab76953), and anti-γ-H2AX (cat. no. ab11174) antibodies were obtained from Abcam (Cambridge, MA, USA), and anti-p21 (cat. no. 556431) and anti-p27 (cat. no. 610242) antibodies were purchased from BD Biosciences (Franklin Lakes, NJ, USA). Anti-p53 antibodies (DO-7; cat. no. SC-47698) and horseradish peroxidase-conjugated anti-mouse and anti-rabbit antibodies were purchased from Santa Cruz Biotechnology (Dallas, TX, USA). Antibodies targeting STING (cat. no. 13647), phospho-signal transducer and activator of transcription 6 (STAT6; cat. no. 56554), phospho-interferon regulatory factor (IRF) 3 (cat. no. 4947), tri-H3K9 (cat. no. 13969), and β-actin (cat. no. 4967S) and secondary antibodies for immunofluorescence were obtained from Cell Signaling Technologies, Inc. (Danvers, MA, USA). siRNA duplexes against human and mouse HMGB1, TRIM30α, STING, STAT6, and p21 and nonspecific control siRNA were purchased from Bioneer Inc. (Daejeon, Korea). Doxorubicin (Dox) was obtained from Calbiochem (San Diego, CA, USA). Actinomycin D, cycloheximide, MG132, and hydrogen peroxide (H_2_O_2_) were purchased from Sigma-Aldrich (St. Louis, MO, USA).

### Cell morphology analysis and SA-β-GAL staining

Morphological changes in cells were photographed using an inverted phase-contrast microscope (Olympus, Tokyo, Japan). SA-β-GAL staining was performed as previously described^18^. Morphological examinations were performed 3 days after each treatment unless otherwise indicated.

### Western blot analysis

Cells were collected and lysed in radioimmunoprecipitation assay buffer containing protease inhibitor cocktail (GenDEPOT, TX, USA). The lysed cells were sonicated and incubated on ice for 30 min. The lysates were centrifuged, and protein concentrations in the cleared lysates were quantified using bicinchoninic acid protein assay reagents (Pierce). Equal amounts of total protein were separated by sodium dodecyl sulfate (SDS) polyacrylamide gel electrophoresis. After the transfer of proteins to nitrocellulose membranes, the membranes were blocked with 5% (w/v) nonfat dry milk in 1× phosphate-buffered saline (PBS) overnight at 4 °C. Proteins were detected with specific antibodies. Antibody–antigen complexes were detected using horseradish peroxidase-conjugated secondary antibodies and visualized using a standard chemiluminescence method performed according to the manufacturer’s instructions.

### Quantitative real-time PCR (qRT-PCR) analysis

RNA was isolated with an RNA extraction kit (iNtRON Biotechnology DR Inc., Daejeon, Korea) and subjected to reverse transcription. qRT-PCR was performed using Power SYBR Green Master Mix (Applied Biosystems, Foster City, CA, USA) with specific primers on a Step One Plus Real-Time PCR System (Applied Biosystems). The relative expression levels of *p21*, *IL-6*, and *IL-8* mRNA were calculated after normalizing the Ct values to that for β-actin expression in the same sample, using the ΔΔCt method.

### Chromatin immunoprecipitation (ChIP) analysis

To examine the presence of HMGB1-binding sites within the TRIM30α promoter region, we performed chromatin immunoprecipitation analysis. To do this, a reporter plasmid was constructed with the TRIM30α promoter region sequence, which corresponds to GRCm38:7: 104464459:104466001 by using the following primers: sense: TTTGCTAGCTTTTTTCAGCTTTCTTGGTATCCAGA, antisense: TTTAAGCTTAGGAGCCACCACACCTGATTTT. Cells were transfected with the constructed plasmid and grown to 90% confluence in two confluent 150-cm^2^ dishes per sample, collected, washed with PBS, and then resuspended with PBS. Next, cells were crosslinked with 0.75% formaldehyde at 25 °C for 10 min, and glycine was added to a final concentration of 125 mM. Cells were then incubated at room temperature for 5 min with gentle shaking. Samples were centrifuged, washed with PBS, resuspended in lysis buffer (1% SDS, 10 mM ethylenediaminetetraacetic acid, 50 mM Tris-HCl [pH 8.1]), and sonicated three times for 15 s each at the maximum setting. Supernatants were then recovered by centrifugation at 12,000 rpm for 10 min at 4 °C, diluted with dilution buffer (0.5% Triton X-100, 2 mM ethylenediaminetetraacetic acid, 100 mM NaCI, 20 mM Tris-HCl [pH 8.1]). Next, a 50 μL sample for input was taken, and purified DNA was used to calculate the DNA concentration. An amount of chromatin equivalent to approximately 25 μg DNA was used for each IP, and 2 μg of the antibody with 20 μL protein G beads (of 50% slurry) was combined and incubated overnight. After washing three times, complexes were eluted by adding 250 μL elution buffer (1% SDS/0.1 M NaHCO_3_) to pelleted beads for reverse cross-linking and centrifuged at 14,000 rpm for 3 min. Pelleted DNA from the supernatants was purified using a spin column, and PCR was then carried out with target-specific primers.

### Luciferase reporter assay

Cells were seeded onto 12-well plates and cotransfected with firefly luciferase reporter fused to the TRIM30α promoter (100 ng) and either an empty vector or a vector expressing HMGB1, SiC, or SiHMGB1. At 3 days after transfection, the lysates were analyzed using luciferase reporter assays (Promega) according to the manufacturer’s protocol.

### Immunofluorescence

Immunofluorescence analysis was performed as previously described^14^.

### Cell counting

Trypan blue solution (0.4%) was added to the cell suspension, and samples were incubated for 5 min at room temperature. Cells without staining were counted using a hemocytometer under a microscope as viable cells^19^.

### Mouse experiment

All animal procedures were approved by the Institutional Animal Care and Use Committee (IRB no. 2019-0242). Briefly, 7- to 8-week-old female C57BL/6 mice were housed in a specific pathogen-free facility and were used for allograft tumor experiments. To generate tumors, 1 × 10^6^ B16-F10 cells suspended in 100 μL PBS were injected into the dorsal subcutaneous area of mice, and tumor masses were successfully formed after implantation. For tumor irradiation (IR) experiments, IR was performed using an X-RAD 320 system (Precision X-Ray Inc., North Branford, CT, USA). The mice were irradiated with a single dose of 10 Gy X-ray. Three hours after the end of the last fraction, the mice were euthanized using CO_2_, and the tumor masses were extracted for subsequent experiments. For the Dox treatment experiment, mice were administered a single intraperitoneal injection of Dox (9 mg/kg body weight) after tumor formation, and tumors were collected 3 or 8 days later.

### Isolation and culture of BMDMs

To generate BMDMs, bone marrow cells were collected from *Trim30*^*+/+*^ and *Trim30*^*−/−*^ mice by flushing with Dulbecco’s PBS. BMDMs were differentiated for 7 days in Dulbecco’s modified Eagle’s medium supplemented with 20% fetal bovine serum, 1% penicillin–streptomycin, 50 U/mL penicillin, and granulocyte macrophage-colony-stimulating factor^20^.

### Statistical analysis

Differences among various experimental groups were evaluated by analysis of variance for multiple comparisons using GraphPad Prism 5 software (GraphPad Software, Inc., La Jolla, CA, USA). Results with *P* values of less than 0.05 were considered statistically significant.

## Results

### STING-induced senescence via p21

**Fig. 1:**
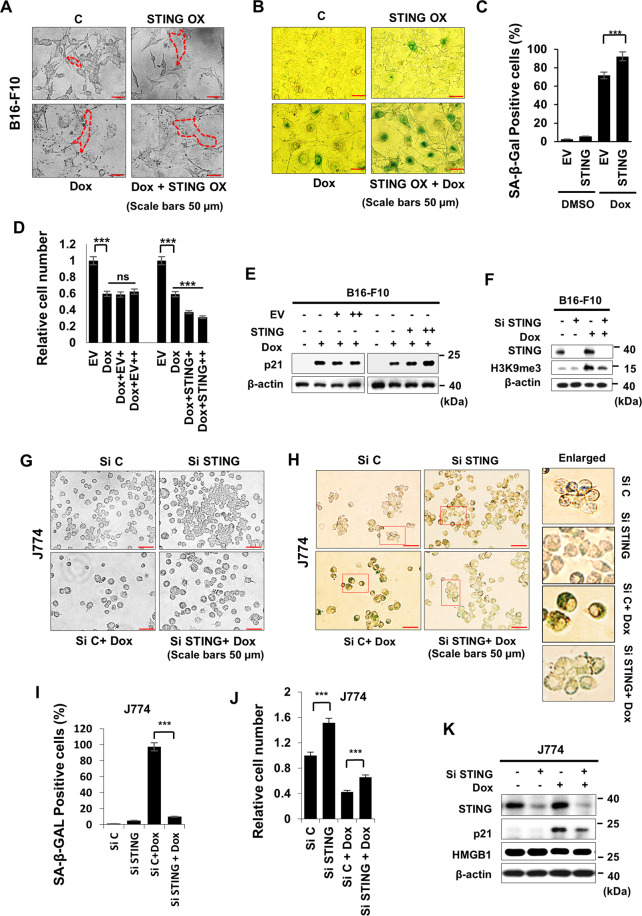
STING-induced senescence. **A**–**C** B16-F10 cells were transfected with empty vector (**C**) or STING plasmid 24 h prior to Dox treatment for 3 days. Cells were photographed (**A**), SA-β-GAL-positive cells (**B**) were counted (**C**). B16-F10 cells were transfected with various concentrations of empty vector (EV) or STING plasmid prior to Dox treatment, and relative cell numbers (**D**) and western blots for p21 (**E**). B16-F10 cells were transfected with Si C or STING prior to 24 h to Dox treatment and Histone 3 lysine 9 trimethylation (H3K9me3) expression was assessed on day 3 (**F**). **G**–**K** J774 cells were transfected with 100 nM of Si C (control) or Si STING 24 h prior to treatment with 100 ng/ml Dox. On day 3 after Dox treatment, cells were photographed (**G**), and SA-β-GAL-positive cells (**H**) were counted (**I**). Relative cell numbers were then quantified (**J**), and western blotting was performed (**K**). Quantitative data are represented as mean ± S.D., *P ≤ 0.05, **P ≤ 0.01, ***P ≤ 0.001, *n* = 3 independent trials Scale bars, 50 μm.

Recent studies have shown that STING is involved in cellular senescence^4,6^. In the present study, the effects of STING overexpression in B16-F10 cells resulted in senescence (Fig. S1A–C), which was demonstrated by various senescence markers, such as p21, γ-H2AX, histone 3 lysine 9 trimethylation (H3K9me3) (Fig. S1A). Furthermore, it was selectively dependent on p21 in a dose- and time-dependent manner, but not on p16 among the CDK inhibitors (Fig. S1B, C). And this effect on senescence was exacerbated in the presence of genotoxic agent doxorubicin (Dox), such as cell size, SA-β-GAL positivity, suppression of cell growth, and increased H3K9me3 expression were observed (Fig. 1A–F). When we evaluated the role of STING in senescence in J774 mouse macrophage cell line, we found that Dox treatment increased cell size and SA-β-GAL positivity and that STING knockdown significantly blocked these effects (Fig. 1G–I). In addition, cell numbers were significantly increased when STING was silenced (Fig. 1J), and STING-knockdown cells showed decreased p21 expression induced by Dox treatment (Fig. 1K). Taken together, these results indicated that STING caused senescence in cancer cells and macrophages by regulating the CDK inhibitor p21, which can be differentiated from previous reports where STING induces senescence via senescence-associated secretory phenotype (SASP)^21^.

### HMGB1-modulated STING expression during senescence

**Fig. 2:**
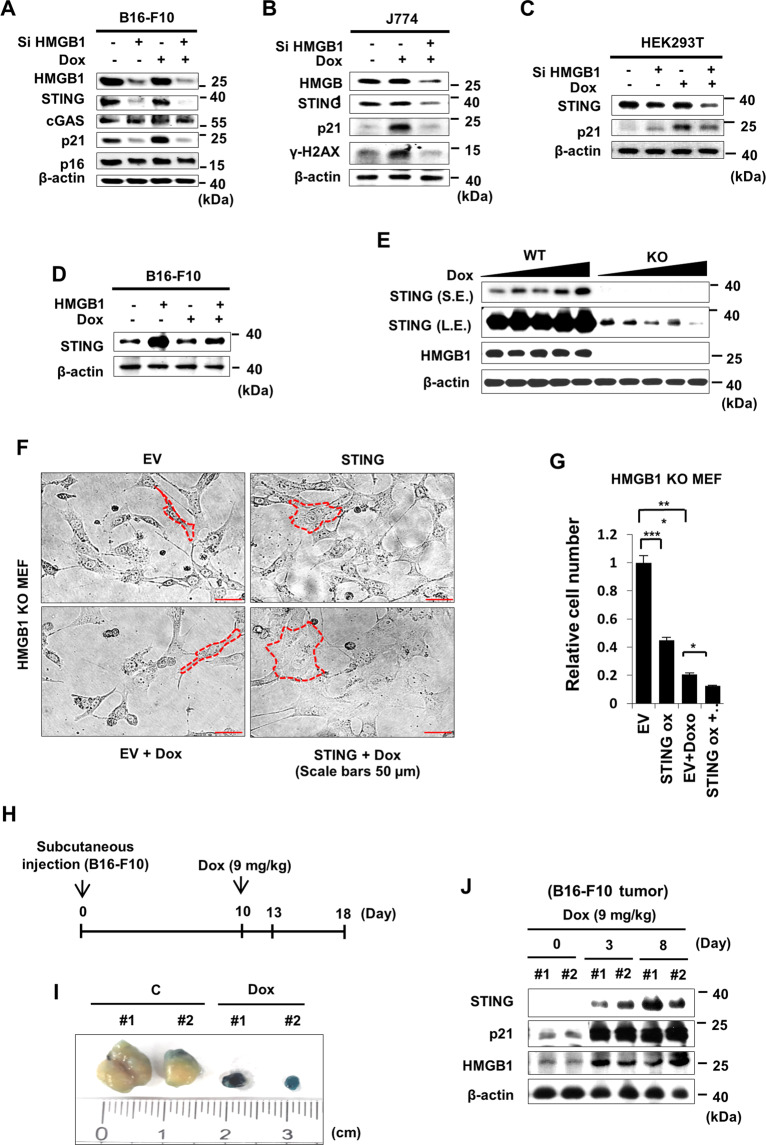
HMGB1-modulated STING during senescence. B16-F10 cells were transfected with 100 nM Si C or Si HMGB1 24 h prior to treatment with 100 ng/mL Dox, and western blotting was performed on day 3 after Dox treatment (**A**). J774 and HEK293T cells were transfected with 100 nM Si HMGB1 24 h prior to treatment with 100 ng/mL Dox, and western blotting was performed on day 3 after Dox treatment (**B**, **C**). B16-F10 cells were transfected with HMGB1 plasmid prior to treatment with 100 ng/mL Dox. Western blotting was then performed on day 3 after Dox treatment (**D**). HMGB1-WT vs. -KO MEFs were treated with Dox, and western blotting was performed (**E**). HMGB1-KO MEFs were transfected with STING plasmid 24 h prior to Dox treatment, then morphological changes (**F**) and relative cell number were assessed on day 3 after Dox treatment (**G**). Tumors were generated by the implantation of B16-F10 cells into C57BL/6 mice. Mice were then injected with 9 mg Dox/kg body weight on day 10. Tumors were collected on days 0, 3, and 8 after Dox treatment (**H**), and tumors were stained with SA-β-GAL staining solution on day 8 after Dox treatment (**I**), following which western blotting was performed (**J**).

Recently, we reported that HMGB1 orchestrated senescence via p21 under the genotoxic stresses in various cancer cell lines^14^. Thus, we aimed to investigate whether HMGB1 interacted with STING to regulate senescence. Accordingly, we evaluated the effects of HMGB1 on STING-induced senescence. First, we employed B16-F10 cells to assess the role of HMGB1 in STING expression followed by Dox treatment. Strikingly, knockdown of HMGB1 significantly reduced STING and p21 expression levels, whereas the expression of cGAS, the upstream component of STING in intracellular DNA sensing, was not changed (Figs. 2A, S2A, B), suggesting that STING and p21 may be specific targets of HMGB1. Similar results for STING expression were observed in J774 and HEK293T cells following HMGB1 knockdown (Fig. 2B, C), and other senescence markers, such as p21 and γ-H2AX were dependent on HMGB1. Overexpression of HMGB1 increased STING expression (Fig. 2D). These results showed that STING was specifically regulated by HMGB1.

Notably, STING expression was completely dependent on HMGB1, as demonstrated in a comparison of HMGB1 wild-type (WT) vs. knockout (KO) MEFs (Fig. 2E). To elucidate whether STING was sufficient to induce senescence without HMGB1, we overexpressed STING in HMGB1-KO MEFs and examined morphological changes and cell numbers. STING overexpression resulted in a senescence-like morphology (Fig. 2F) and reduced cell numbers, potentially because of the induction of senescence (Fig. 2G).

Because anticancer drug treatment is known to induce senescence in cancer cells^1,2^, we then generated melanoma tumor models in mice and treated the mice with Dox (Fig. 2H). Dox-treated tumors were positive for SA-β-GAL staining (Fig. 2I). Indeed, the levels of STING and p21 from tumors were correlated with those of HMGB1 following treatment with Dox (Fig. 2J). Taken together, these findings showed that HMGB1 selectively regulated STING expression, which was indispensable for the induction of senescence following Dox treatment, but not upstream or downstream molecule of canonical cGAS/STING factors.

### TRIM30α targeting induced senescence via STING

**Fig. 3:**
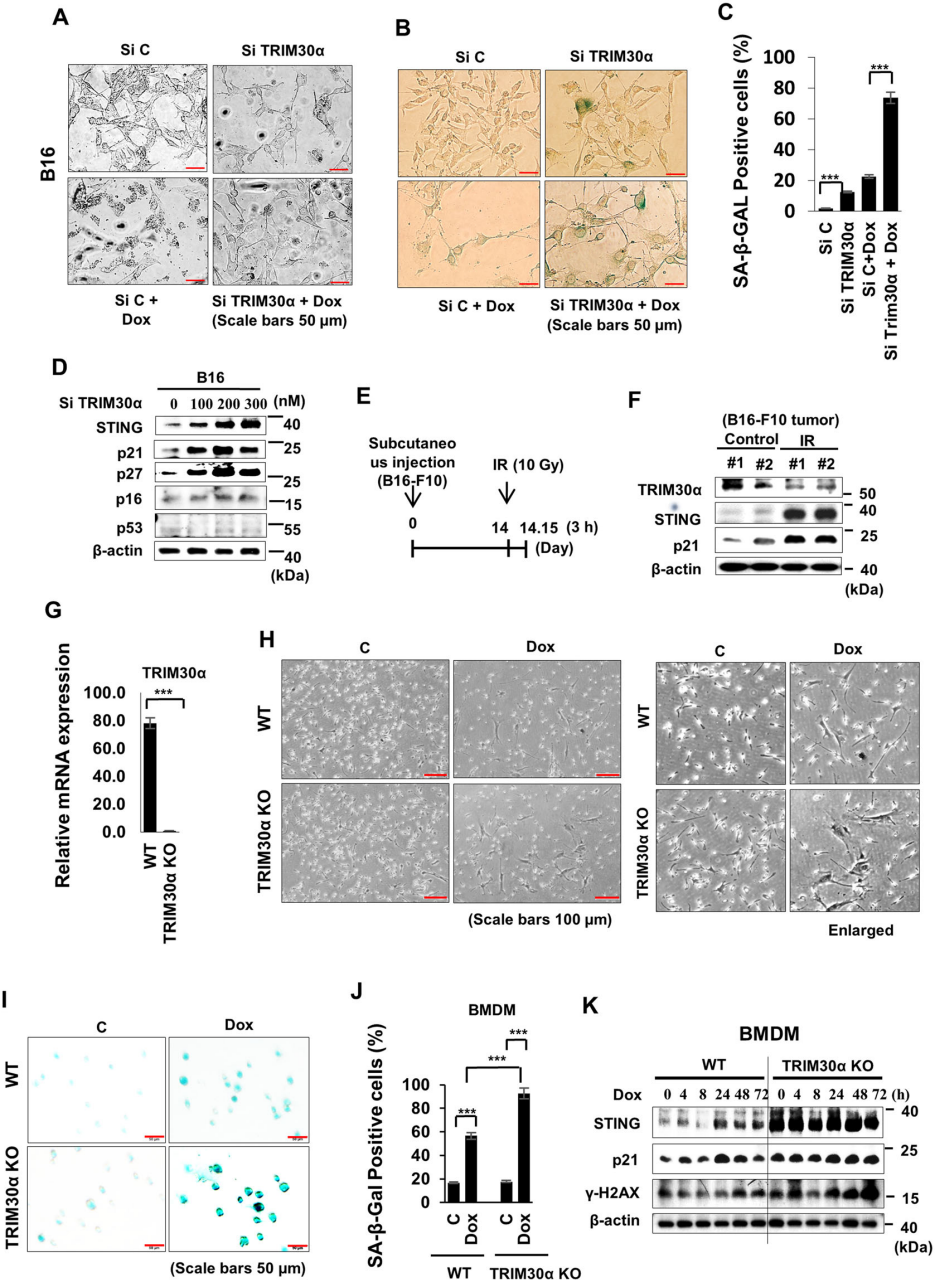
TRIM30α targeting induced senescence via STING. **A**–**C** B16 cells were transfected with Si C or Si TRIM30α 24 h prior to Dox treatment for 3 days. Cells were photographed (**A**), and SA-β-GAL-positive cells (**B**) were counted (**C**). B16 Cells were transfected with various concentrations of Si TRIM30α, and western blotting was performed on day 3 after Dox treatment (**D**). **E**, **F** Tumors were generated by implantation of B16-F10 cells into mice. On day 14 after implantation, tumors were exposed to IR. Three hours later, tumors were harvested and analyzed by western blotting. **G**–**K** TRIM30α WT and KO mouse BMDMs were employed for analyzing the effects of TRIM30 on senescence. Real-time PCR of TRIM30α was performed with TRIM30α-WT and -KO BMDMs (**G**). TRIM30α-WT and -KO BMDMs were treated with 100 ng/mL Dox, and morphological changes (**H**: left, enlarged images: right), images (**I**), and counts (**J**) of SA-β-GAL-positive cells were evaluated on day 3 after treatment. Western blotting was performed at the indicated times after treatment with 100 ng/mL Dox (**K**).

TRIM30α, an E3 ligase of the TRIM family in mice^22^, is responsible for STING degradation^23^. Therefore, we next investigated whether TRIM30α affected STING regulation in senescence, although little has been known of the role of TRIM30α in senescence. Because mouse melanoma B16 cells could not enter senescence following Dox treatment^14^, presumably due to the lack of sufficient STING expression, we knocked down TRIM30α and then treated cells with Dox to protect STING from degradation. Interestingly, TRIM30α knockdown alone forced cells to enter a senescent state, as observed by morphological changes (Fig. 3A), and promoted senescence following Dox treatment by SA-β-GAL-positive morphology and cell numbers (Fig. 3B, C). In fact, STING expression was increased in a Si TRIM30α concentration-dependent manner, which was accompanied by increased expression of the CDK inhibitors p21 and p27, but not p16 or p53 (Fig. 3D).

Next, we established a tumor model generated by implanting B16-F10 melanoma cells into C57BL/6 mice and treated the tumor regions with IR. Indeed, although STING and p21 were significantly upregulated in IR-treated tumors, TRIM30α expression showed the opposite trend (Fig. 3E, F). We also hired J774 cells to examine whether these phenomena were observed in mouse macrophage cells as well (Fig. S3A–E). When J774 cells were treated with Si TRIM30α followed by Dox, TRIM30α knockdown enhanced the effects of Dox on the senescence phenotype, including morphological changes (Fig. S3A), decreased cell numbers (Fig. S3B), increased SA-β-GAL-positive cell numbers (Fig. S3C, D), and STING upregulation (Fig. S3E). Finally, we expended these experiments to TRIM30α-KO mouse bone marrow-derived macrophages (BMDMs) to verify the function of TRIM30α in senescence (Fig. 3G). Indeed, the effects of Dox treatment were enhanced in TRIM30α-KO mouse BMDMs as shown in the senescence phenotype as demonstrated by increased cell size (Fig. 3H), SA-β-GAL positivity (Fig. 3I, J), and STING, p21, and γ-H2AX expression (Fig. 3K). Taken together, these findings supported that TRIM30α targeting was critical for inducing senescence by preventing STING degradation.

### HMGB1-modulated TRIM30α expression to induce senescence

**Fig. 4:**
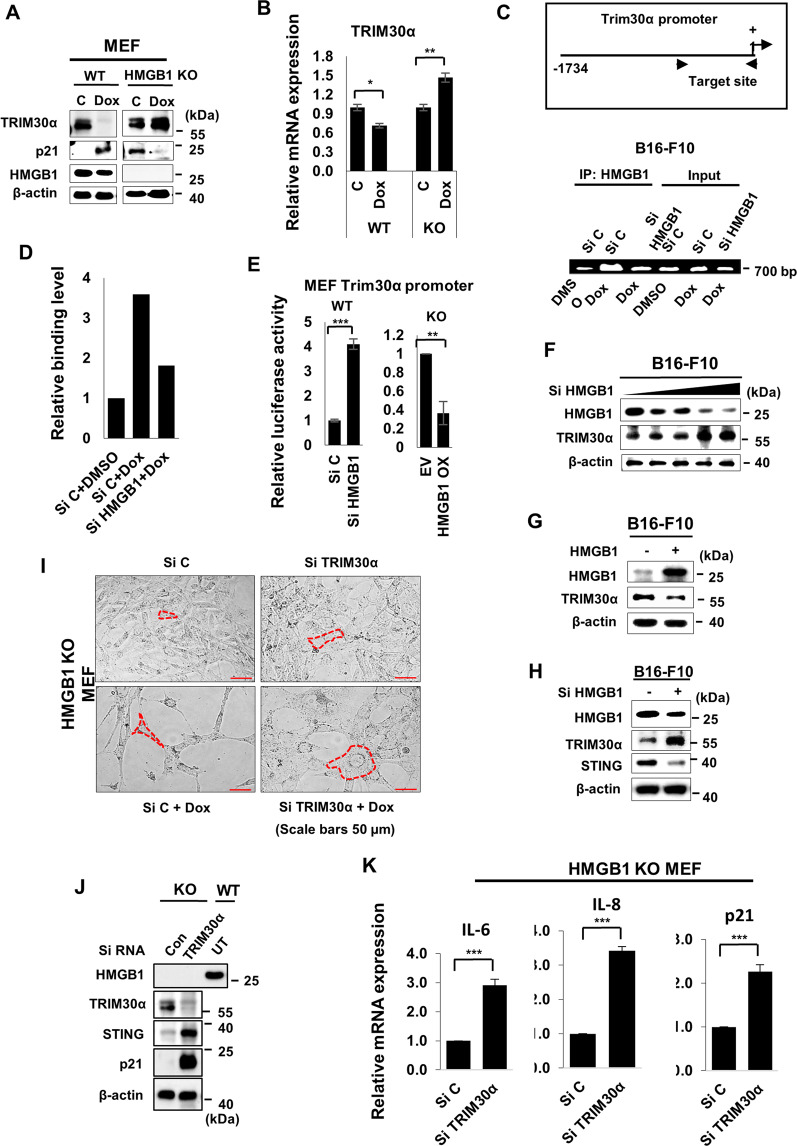
HMGB1-modulated TRIM30α to induce senescence. HMGB1-WT and -KO MEFs were treated with 100 ng/ml Dox for 3 days, then western blotting (**A**) and qPCR (**B**) were performed to assess TRIM30α expression. Cells were transfected with Si C or Si HMGB1 24 h prior to Dox treatment to B16-F10 cells for 3 days, and immunoprecipitation was performed with anti-HMGB1 antibodies. PCR was carried out using TRIM30α promoter-specific primers for ChIP assays (**C**), and the binding level was quantified (**D**). TRIM30α promoter activity was measured by luciferase assay at day 3 after Dox treatment (**E**). Cells were transfected with Si HMGB1 in a concentration-dependent manner for 2 days, and western blotting was performed (**F**). Cells were transfected with HMGB1 plasmid (**G**) or Si HMGB1 (**H**) for 2 days, and western blotting was performed. HMGB1-KO MEFs were transfected with 100 nM Si C or Si TRIM30α for 3 days. Morphological changes were photographed (**I**), protein expression was evaluated by western blotting (**J**), and mRNA expression was estimated by qPCR (**K**). Quantitative data are represented as mean ± S.D., *P ≤ 0.05, **P ≤ 0.01, ***P ≤ 0.001, *n* = 3 independent trials Scale bars, 50 μm.

Due to the effects of TRIM30α on STING-dependent suppression of senescence, we investigated the role of TRIM30α in HMGB1-mediated senescence. Because STING was positively regulated by HMGB1 (Fig. 2), we hypothesized that TRIM30α may be negatively modulated by HMGB1 in senescence. First, we treated HMGB1-WT and -KO MEFs with Dox and then assessed TRIM30α expression. Interestingly, TRIM30α protein expression was markedly reduced in WT MEFs but was upregulated in KO MEFs following Dox treatment; the opposite expression pattern was observed for p21 (Fig. 4A). Changes in TRIM30α mRNA expression were consistent with changes in protein expression (Fig. 4B). These findings suggested that the high expression of TRIM30α was responsible for blocking the entry of KO MEFs into a senescent state by Dox treatment. For further investigating the effects of HMGB1 on TRIM30α, we performed ChIP assays, the results of which showed that HMGB1 bound to the promoter region of TRIM30α; the binding affinity was much higher in Dox-treated conditions but decreased following silencing of HMGB1 (Fig. 4C, D). In addition, TRIM30α promoter activity was inversely correlated with HMGB1 expression in MEFs (Fig. 4E). Similarly, TRIM30α protein expression showed an inverse correlation with HMGB1 expression in an HMGB1-silencing dose-dependent manner in B16-F10 cells (Fig. 4F), and these findings were further confirmed by the upregulation or downregulation of HMGB1 (Fig. 4G, H). Furthermore, these were also supported in HMGB1-WT and -KO MEFs (Fig. S4).

Next, we assessed the effects of TRIM30α knockdown in KO MEFs (Fig. 4I–K). Si TRIM30α transfection alone and Si TRIM30α transfection plus Dox treatment resulted in a senescence-like morphology in cells (Fig. 4I). In addition, TRIM30α depletion increased STING and p21 protein expression (Fig. 4J) and enhanced the mRNA levels of senescence markers, including *IL-6*, *IL-8*, and *p21*, in KO cells (Fig. 4K). Collectively, these findings suggested that HMGB1 controlled TRIM30α expression to maintain STING for inducing senescence.

### STAT6-regulated HMGB-mediated senescence

**Fig. 5:**
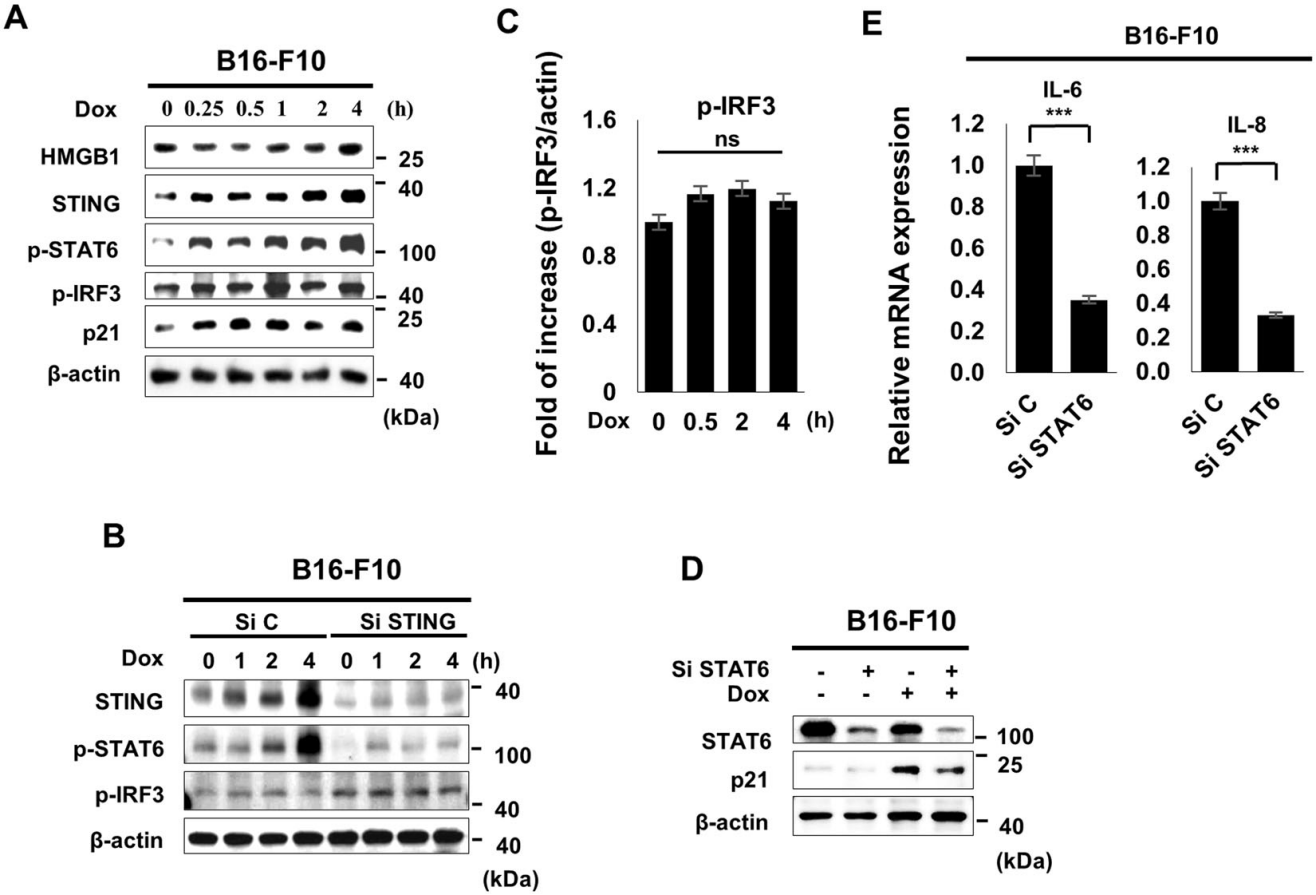
STAT6-mediated HMGB1-dependent senescence. B16-F10 cells were treated with Dox in a time-dependent manner, and western blotting was performed with the indicated antibodies (**A**). B16-F10 cells were transfected with Si C or Si STING 24 h prior to Dox treatment for indicated times, and western blotting was performed (**B**). Phospho-IRF3 expression levels were assessed at the indicated times (**C**). B16-F10 cells were transfected with Si STAT6 24 h prior to Dox treatment for the indicated times, and western blots were assessed on day 3 after Dox treatment (**D**). Relative mRNA expression levels of IL-6 and IL-8 were evaluated on day 3 after Si STAT6 transfection (**E**). Quantitative data are represented as mean ± S.D., *P ≤ 0.05, **P ≤ 0.01, ***P ≤ 0.001, *n* = 3 independent trials Scale bars, 50 μm.

**Fig. 6:**
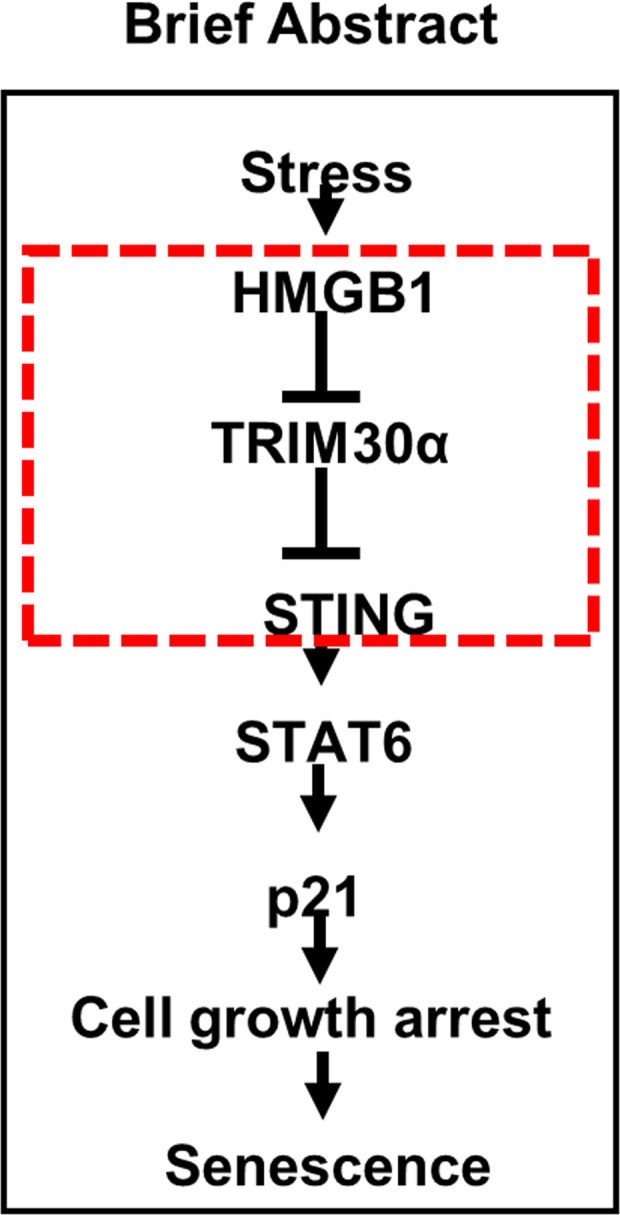
Proposed mechanism of HMGB1-mediated senescence induced by genotoxic stress. The dotted box shows our key findings from the current study.

STAT6 acts downstream of STING as a transcription factor of p21. STING recruits STAT6 to the ER, and TBK then induces STAT6 phosphorylation, resulting in translocation of STAT6 into the nucleus and facilitating the induction of target genes^24^. In addition, in human breast cancer cells, STAT6 functions in G_1_/S cell cycle progression, and the growth-inhibitory effects of STAT6 are mediated by induction of the G1 CDK inhibitors p21^Cip1/WAF1^ (p21) and p27^Kip1^ (p27)^25^. As expected, phospho-STAT6 levels were dramatically increased along with STING, p21, and HMGB1 expression following Dox treatment at early time points (Fig. 5A); this effect could be significantly blocked by STING silencing (Fig. 5B). However, phospho-IRF3, another canonical mediator of STING signaling, was not significantly altered by Dox and STING silencing (Fig. 5A–C). In addition, p21 protein and SASPs *IL-6* and *IL-8* mRNA expression levels were downregulated by STAT6 silencing (Fig. 5D, E). Taken together, these findings indicated that STAT6 may be a specific target of STING in HMGB1-mediated senescence. The proposed mechanism is presented in Fig. 6.

## Discussion

Senescence has emerged as an alternative anticancer mechanism to apoptosis in chemotherapy and IR treatment^26,27^. HMGB1 is involved in various diseases, including cancer and senescence^14,16,28^. In our previous study, we identified HMGB1 as a critical determinant of cancer cell fate. Particularly, cancer cells underwent senescence selectively rather than apoptosis depending on HMGB1 expression. HMGB1 induces senescence via the CDK inhibitor p21 in the context of genotoxic stresses, such as Dox, camptothecin, and IR, and this mechanism could be applicable to drug-resistant cancer cells therapy^14^.

STING positively regulates senescence via the cGAS/STING pathway^4,6^. In addition, STING can act independently of cGAS^29–31^. Senescence orchestrated by the cGAS/STING pathway occurs through the SASP rather than the canonical senescence pathway, i.e., the p53/p21 or p16^INK4a^/Rb pathway^21^. Similarly, HMGB1 and STING have separate roles in senescence induced by various stimuli, including chemotherapeutic agents; however, no studies have examined whether these two proteins interact during senescence. Therefore, in this study, we evaluated the roles and mechanisms of STING in HMGB1-mediated senescence, using B16-F10 mouse melanoma cells, which were reported to undergo senescence through a mechanism involving HMGB1/p21 in the presence of genotoxic stress^14^, and various cell lines including MEFs and macrophages.

In our previous study, we demonstrated that HMGB1 is indispensable for inducing senescence. In the present study, we found that STING, but not cGAS, was regulated by HMGB1 to induce senescence. Interestingly, STING selectively regulated p21, but not other CDK inhibitors, such as p16, to cause cell cycle arrest in senescence. Furthermore, among known STING downstream signals, STAT6 was selectively activated to induce p21 in our study^24,25,32^.

Most TRIM family proteins have E3 ubiquitin ligase activities and function in protein quality control, innate immunity, apoptosis, autophagy, carcinogenesis, intracellular signaling, and development^22^. However, the role of TRIM30, specifically TRIM30α in senescence is still unclear. TRIM30α is a negative regulator of STING^23^. Notably, in this study, we found that TRIM30α suppressed STING expression to prevent senescence and that TRIM30α silencing upregulated STING and p21, which were required for induction of senescence. In addition, TRIM30α was significantly downregulated in senescent WT MEFs but was upregulated in HMGB1-KO MEFs treated with Dox. In our study, we showed that HMGB1 negatively regulated TRIM30α in senescent WT MEFs induced by Dox, which contributed to senescence via STING and p21 upregulation. Therefore, we concluded that HMGB1 suppressed TRIM30α transcription by binding to its promoter region, and this effect was blocked by HMGB1 silencing. Thus, our findings highlighted the involvement of TRIM30α in the HMGB1/STING-mediated senescence pathway.

To confirm our findings in vivo, we established B16-F10 cell-derived tumors in mice and evaluated the effects of Dox and IR on these tumors. Notably, Dox- and IR-treated tumors showed significant upregulation of STING and p21 in the presence of HMGB1, whereas TRIM30α was downregulated; these findings strongly supported our hypothesis. However, additional studies are required to further elucidate the function of the HMGB1/STING axis in other pathologies.

Cellular senescence can be detected in immune cells. Immunosenescence is a slightly different process from cellular senescence. Immunosenescence is a cellular hypoproliferation response to mitogenic or antigen stimulation and is often observed in aging immune cells. Although the molecular determinants of immunosenescence have not been very well described, similarities with senescence in nonimmune cells are found. For example, the immunosenescence of T cells is regulated by p16^INK4A^ and p21^CIP1 ^^33,34^. In our study, J774 mouse macrophages also shared premature senescence mechanisms of cancer cells induced by a chemotherapeutic agent.

In summary, our findings supported that senescence may be an alternative anticancer mechanism and that HMGB1 selectively functioned via a pathway involving STING/STAT6/p21 to induce senescence. This pathway involved suppression of TRIM30α expression by HMGB1 to promote STING-mediated senescence in the context of genotoxic stress (Fig. 6). To the best of our knowledge, this is the first study to report a link between HMGB1 and STING in senescence. Furthermore, although most studies on STING have shown that STING modulates senescence via the canonical cGAS/STING pathway, in this study, we demonstrated an unconventional mechanism of STING-mediated senescence, independent of cGAS, in cancer cells. Our findings could contribute to cancer prevention via modulation of the HMGB1/TRIM30α/STING pathway to suppress tumor cell growth by inducing senescence. Further studies are needed to investigate whether these mechanisms can be applied to other types of cancer cells and tissues.

## Supplementary information

Supplementary figure legends

Table

Supplementary Figure 1.

Supplementary Figure 2.

Supplementary Figure 3.

Supplementary Figure 4.
