# Supplementary material for: HMGB1 orchestrates STING-mediated senescence via TRIM30α modulation in cancer cells
Source: Cell Death Discov. 2021 Feb 8;7:28. doi: 10.1038/s41420-021-00409-z (PMC7870821; doi:10.1038/s41420-021-00409-z)
Supplement: Supplementary file 1 — Supplementary figure legends [file 41420_2021_409_MOESM1_ESM.docx]

**Supplemental Figure 1.**  **STING induced senescence via p21.** B16-F10 cells were transfected with only EV or STING plasmids, and western blotting for H3K9 and γ-H2AX (A), p21, p16 expression in STING dose dependent- and time dependent-manner were observed (B-C).

**Supplemental Figure 2. HMGB1 modulated STING expression during senescence. Figure 2. HMGB1 modulated STING during senescence.** B16-F10 cells were transfected with 100 nM Si C or two distinct Si HMGB1, and western blotting was performed on day 3 after Dox treatment (A). B16-F10 cells were transfected with 100 nM Si C or Si HMGB1 24 h prior to treatment with 100 ng/mL Dox, and western blotting was performed on day 3 after Dox treatment (B).

**Supplemental Figure 3.**  **TRIM30α induced senescence via STING in J774 cells**. J774 cells were transfected with 100 nM of Si C (control) or Si TRIM30α 24 h prior to treatment with 100 ng/ml Dox. On day 3 after Dox treatment, cells were photographed (A), relative cell numbers were then quantified (B), and SA-β-GAL-positive cells (C) were counted (D), and western blotting was performed (E). Quantitative data are represented as mean ± S.D., *P ≤ 0.05, **P ≤ 0.01, ***P ≤ 0.001, n =3 independent trials Scale bars, 50 μm.

**Supplemental Figure 4. HMGB1 modulated TRIM30α expression to induce senescence.** Western blotting was performed with HMGB1-WT and -KO MEFs.

**Table 1. Summary of siRNA sequences and primer sequences used in experiments.**
