# Supplementary material for: HMGB1 orchestrates STING-mediated senescence via TRIM30α modulation in cancer cells
Source: Cell Death Discov. 2021 Feb 8;7:28. doi: 10.1038/s41420-021-00409-z (PMC7870821; doi:10.1038/s41420-021-00409-z)
Supplement: Supplementary file 2 — Table [file 41420_2021_409_MOESM2_ESM.docx]

**Summary of SiRNA and qPCR primer sequences used in Experiments.**

| **Mouse SiRNA** |  |
| --- | --- |
| **Si HMGB1(m)** | Sense: UGCUGCCUACAGAGCUAAA |
|  | AntiSense: UUUAGCUCUGUAGGCAGCA |
| **Si TRIM30a(m)** | Sense: CUGCGGUGCUCUUCAUCUAU |
|  | AntiSense: AUAGAUGAAGAGCACCGCAG |
| **Si STING (m)** | Sense: GGAUCCGAAUGUUCAAUCA |
|  | AntiSense : UGAUUGAACAUUCGGAUCC |
| **Si Control** | Sense: CCUACGCCACCAAUUUCGU |
|  | AntiSense : ACGAAAUUGGUGGCGUAGG |
| **Si p21(m)** | Sense: AGACCAGCCUGACAGAUUU |
|  | AntiSense: AAAUCUGUCAGG UGGUCU |
|  |  |
|  |  |
| **Mouse qPCR primers** |  |
| p21 qPCR primer(m)-F | GTGGGTCTGACTCCAGCCC |
| p21 qPCR primer(m)-R | CCTTCTCGTGAGACGCTTAC |
| F-b-actin-qPCR(m) | CGCCACCAGTTCGCCATGGA |
| R-b-actin-qPCR(m) | TACAGCCCGGGGAGCATCGT |
| TRIM30a(m)-qPCR-F | TCCAGAGGAGGAGCAGAAGGT |
| TRIM30a(m)-qPCR-R | CCTACAGAAGAGCCGGAGTTTC |
| m IL6-F qPCR | CTTCCATCCAGTTGCCTTCTTG |
| m IL6-R qPCR | AATTAAGCCTCCGACTTGTGAAG |
| m IL8-F qPCR | AAGGCTGGTCCATGCTCC |
| m IL8-R qPCR | TGCTATCACTTCCTTTCTGTTGC |
|  |  |
| **Chip assay primers** |  |
| TRIM30α promoter primer F | TTTGCTCCTCTCCACAGATC |
| TRIM30α promoter primer R | CCTCCAACACACTAAATGCACC |
