# Supplementary figures and images for: HMGB1 orchestrates STING-mediated senescence via TRIM30α modulation in cancer cells
Source: Cell Death Discov. 2021 Feb 8;7:28. doi: 10.1038/s41420-021-00409-z (PMC7870821; doi:10.1038/s41420-021-00409-z)

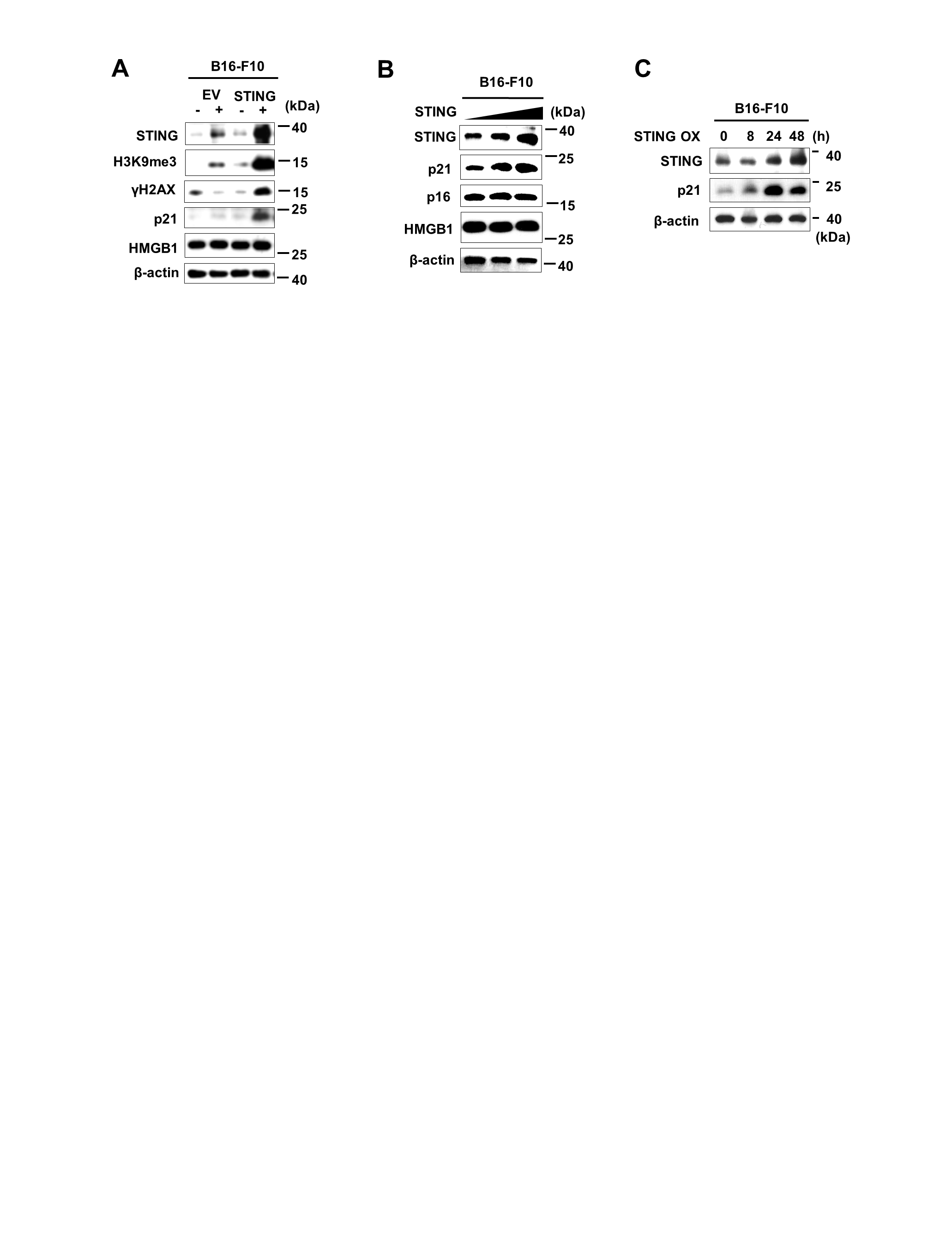

Supplement: Supplementary file 3 — Supplementary Figure 1. [file 41420_2021_409_MOESM3_ESM.tif]

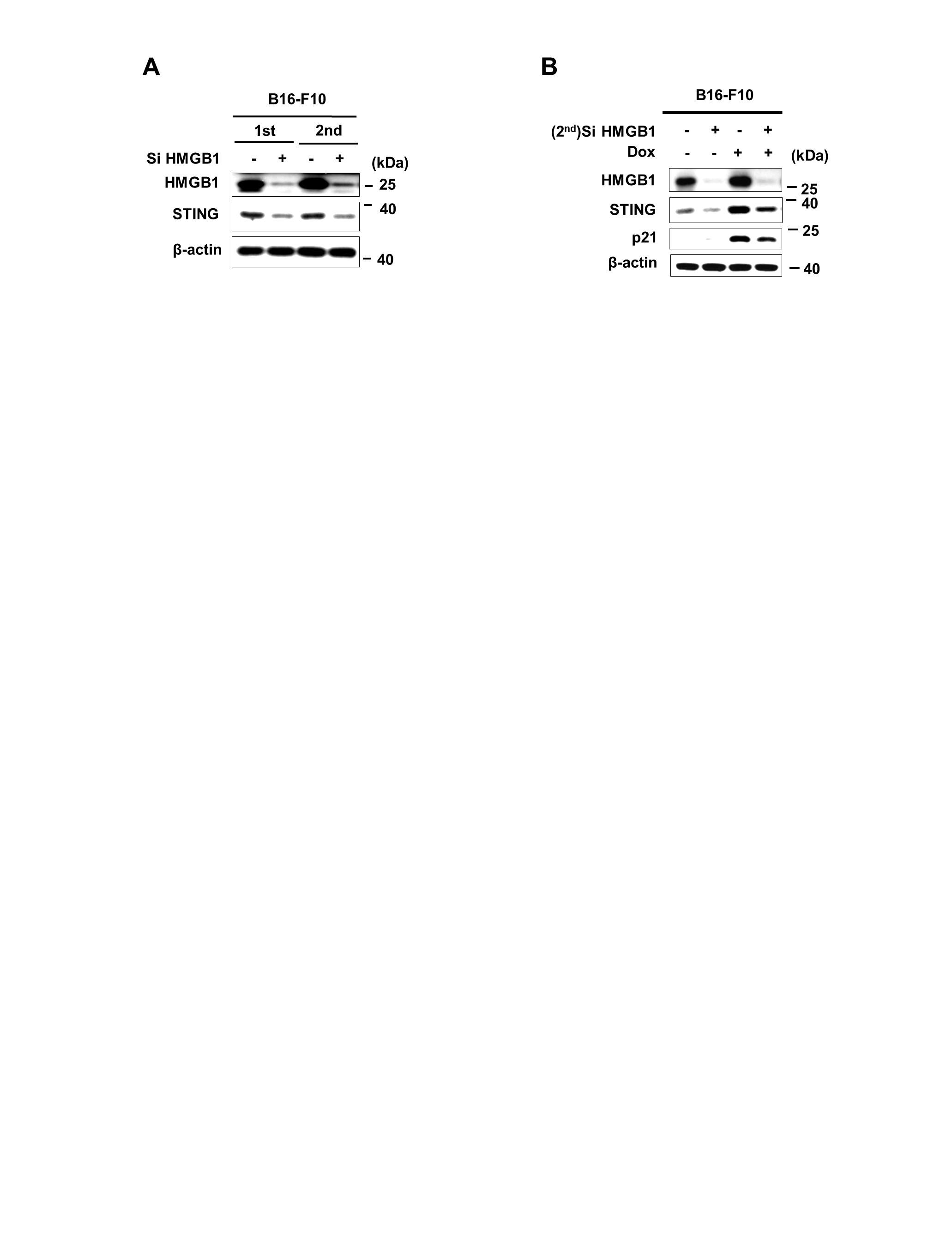

Supplement: Supplementary file 4 — Supplementary Figure 2. [file 41420_2021_409_MOESM4_ESM.tif]

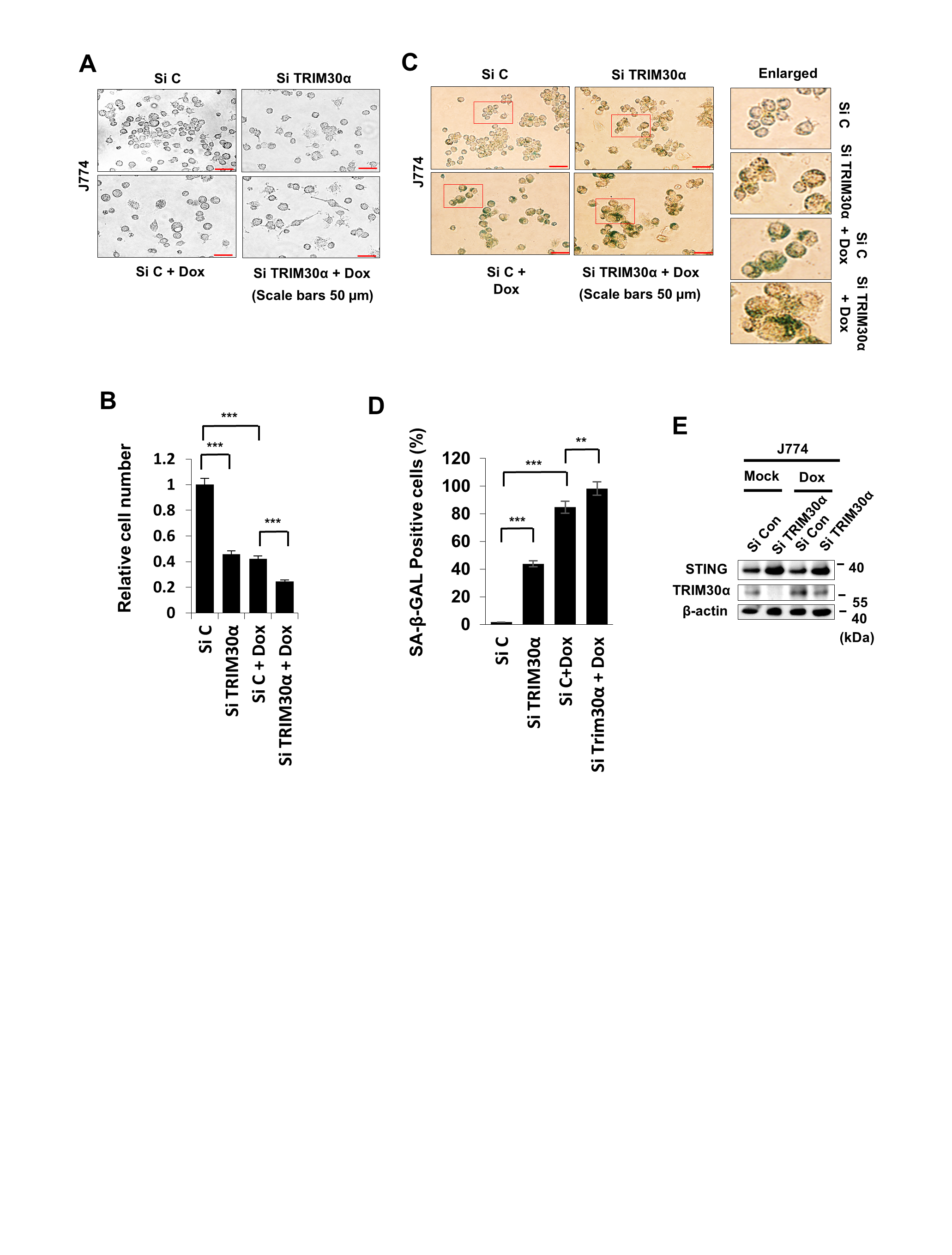

Supplement: Supplementary file 5 — Supplementary Figure 3. [file 41420_2021_409_MOESM5_ESM.tif]

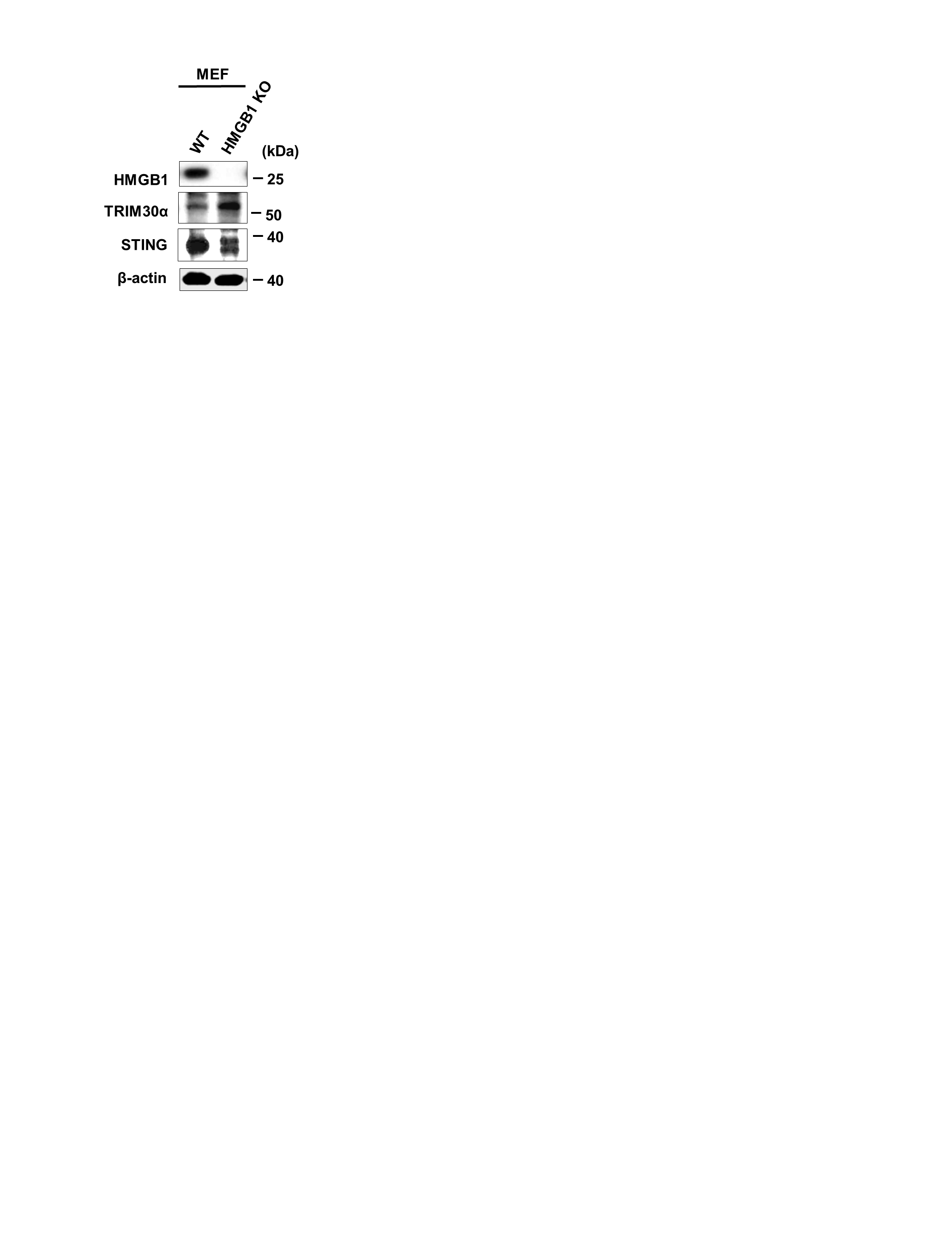

Supplement: Supplementary file 6 — Supplementary Figure 4. [file 41420_2021_409_MOESM6_ESM.tif]
